# Adenocarcinoma of the Stomach With Situs Inversus Totalis: A Rare Case

**DOI:** 10.7759/cureus.31538

**Published:** 2022-11-15

**Authors:** Yashwant Lamture, Pankaj Gharde, Varsha Gajbhiye, Tushar Nagtode, Kiran Mastud, Varun Kulkarni, Dhaval Patel

**Affiliations:** 1 General Surgery, Jawaharlal Nehru Medical College, Datta Meghe Institute of Medical Sciences, Wardha, IND; 2 Pharmacology, Jawaharlal Nehru Medical College, Datta Meghe Institute of Medical Sciences, Wardha, IND

**Keywords:** chemotherapy, gastrectomy, stomach carcinogenesis, metastatic, cancer, gastric adenocarcinoma

## Abstract

Situs inversus is a scarce congenital anomaly. Situs inversus totalis (SIT) is a mirroring of the normal. Thoracic and abdominal viscera transposition is a characteristic feature of situs inversus. It is considered to be a premalignant condition. This uncommon genetic disorder is often identified incidentally during thoracic and abdominal imaging. The coexistence of SIT and gastric cancer is rare. Because this anomaly is known to have associated anatomical and vascular anomalies, due care is required to identify it preoperatively and during the surgical procedure. At centers with prior experience, consistent with oncological practices, open surgeries, laparoscopic surgeries, and robotic surgeries can be done.

We present a patient with a stomach adenocarcinoma with SIT who underwent distal gastrectomy with gastrojejunostomy along with resection and anastomosis of the transverse colon and capecitabin-oxaliplatin chemotherapy. The postoperative course was favorable. To our knowledge, only 13 cases of diffuse-type gastric cancer in a patient with SIT have been reported in the English-language literature.

## Introduction

Gastric adenocarcinoma (GAC) is the 15th most common cause of cancer mortality in males and the third most common cause of cancer death globally [1]. Gastric cancer was once quite common, and due to its poor prognosis, it was one of the deadliest kinds of cancer. The frequency of stomach cancer has decreased drastically during the previous few decades [2]. Because of variations in the biology of GAC, it is not viable to apply single oncological concepts to all stomach cancers [3]. When unusual anatomic abnormalities such as intestinal malrotation and situs inversus occur, proper preoperative identification is imperative. Situs inversus totalis (SIT) is an uncommon anomaly due to a mono-recessive gene leading to a reversal of abdominal organs from their usual locations [4].

Situs inversus is a rare condition with an incidence of one in 10,000 live births. The male gender is affected more commonly than females. Aristotle was the first to describe this condition in animals. By the start of the 16th century, Fabricius identified the first human case of transposition of the liver and spleen. After 50 years of this discovery, Riolan, principal of the University of France, described two patients. Marco Severino is credited with the identification of the first case of dextrocardia in the early 16th century [2]. Küchenmeister claimed to have reported four living subjects. He diagnosed all with his clinical examination skills. He described these anatomical diagrams as situs viscerum transversus [4]. Vehsemeyer was the first to identify this anomaly with the use of radiological investigation such as X-rays in the late 19th century. After this breakthrough, radiology became the preferred investigation to diagnose situs inversus [5].

Allen described the case of a 30-year-old male having SIT with malignancy of the gastric region in the prepyloric area who expired a few weeks postoperatively. Here, we present a case with situs inversus with gastric malignancy who underwent gastric resection by an open method. In the early 2000s, synchronous presentation of situs inversus and stomach malignancy managed laparoscopically was documented first. In 2012, a patient with the synchronous finding of situs inversus and stomach malignancy was operated on with robotic assistance for a partial gastrectomy procedure [6]. In the literature published so far, 32 patients with stomach malignancy have been diagnosed with situs inversus synchronously. Out of these 32 cases, only 12 had diffuse-type histology. Hence, the present diffuse type of gastric cancer will be the 13th case [7].

The coexistence of SIT and gastric cancer is rare. As the condition is associated with anatomical and vascular anomalies, detailed preoperative imaging is important to embark on definitive surgery such as gastric resection.

## Case presentation

**Figure 1:**
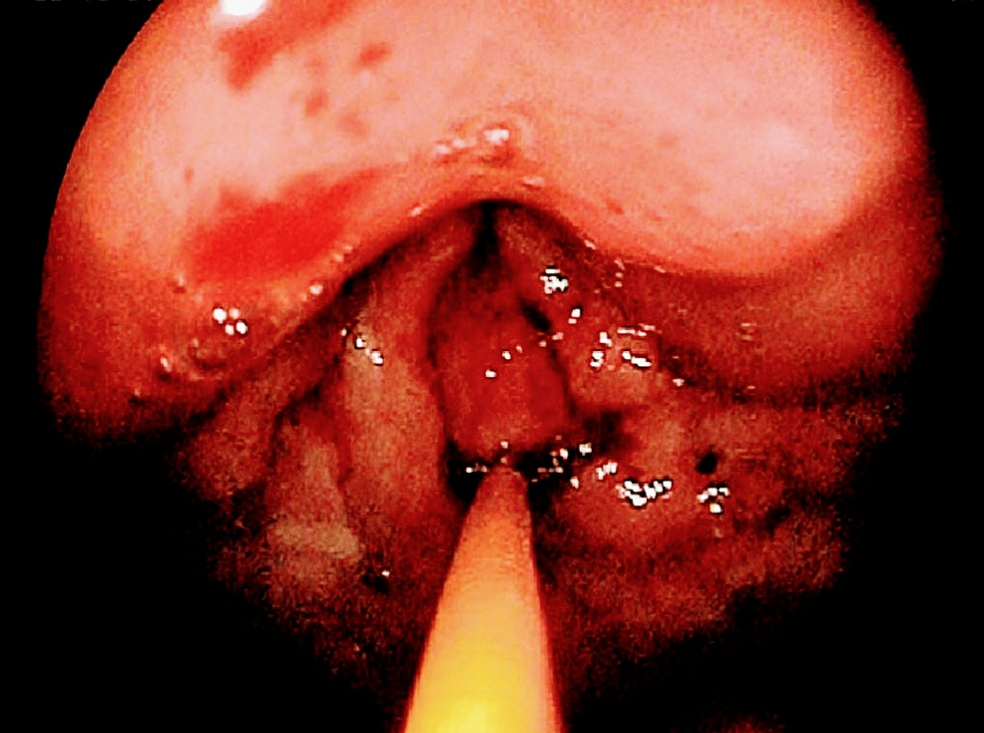
Upper gastrointestinal endoscopy showing evidence of friable proliferative growth at the pylorus occluding complete lumen, suggestive of gastric outlet obstruction.

**Figure 2:**
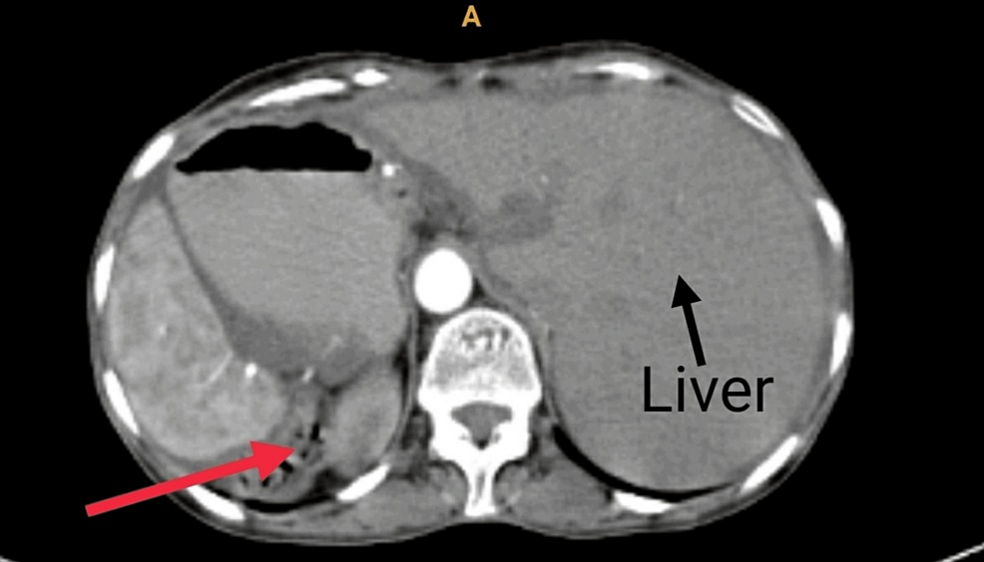
Contrast-enhanced computed tomography of the abdomen. The red arrow shows a thickening of the pyloric wall suggestive of neoplasm, and the black arrow shows the liver on the left side of the abdomen. There is no evidence of metastatic lesions in the liver or involvement of para-aortic lymph nodes.

A 48-year-old female visited the outpatient department with a complaint of severe pain in the lower abdomen for 20 days which was progressive. A history of vomiting was also present for 15 days. A careful physical examination revealed evidence of dextrocardia. The physician advised upper gastrointestinal endoscopy (UGE) and contrast-enhanced computed tomography (CECT) of the abdomen, along with other supportive blood investigations. UGE showed evidence of friable proliferative growth at the pylorus occluding total lumen, suggestive of gastric outlet obstruction (Figure 1). Multiple biopsies were taken which confirmed a diffuse type of well-differentiated adenocarcinoma. On CECT of the abdomen, the stomach appeared malpositioned, suggestive of SIT (Figure 2). It also revealed a thickening of the pyloric wall, suggestive of neoplasm, causing gastric outlet obstruction.

**Figure 3:**
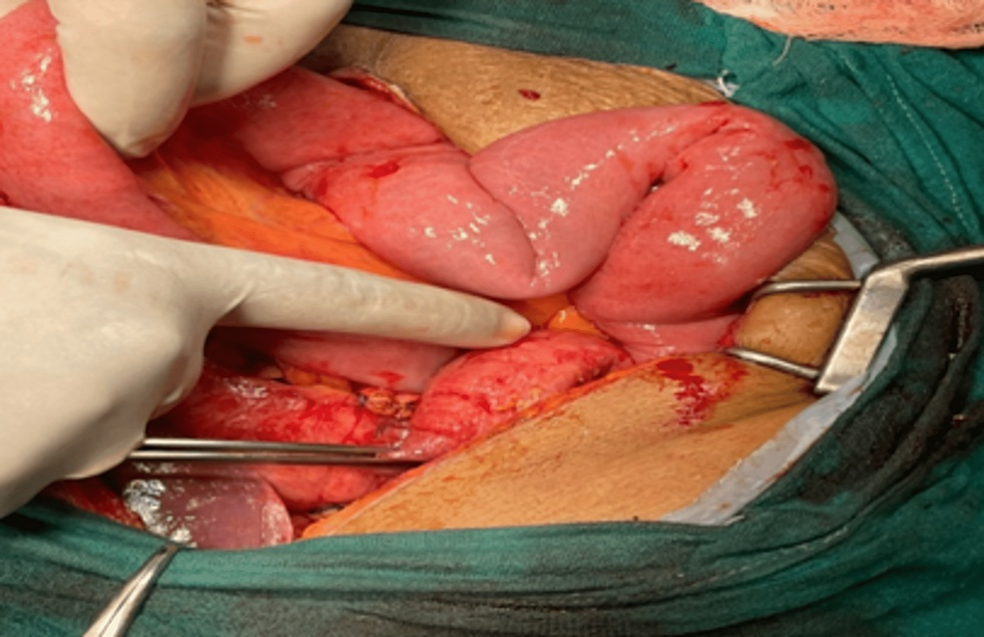
Image showing the pyloric mass (index finger).

**Figure 4:**
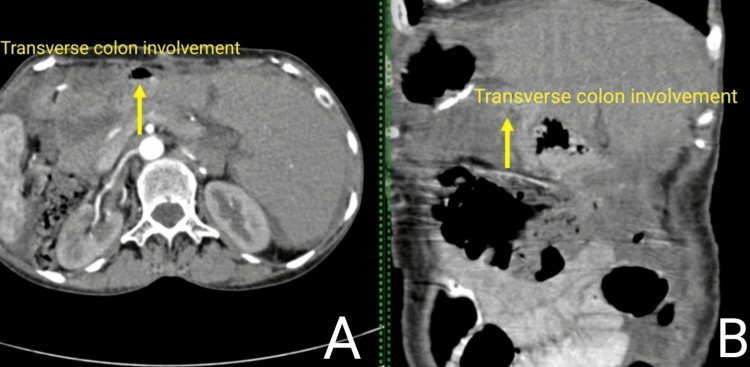
Contrast-enhanced computed tomography (CECT) scan showing involvement of the transverse colon. CECT scan of the abdomen and pelvis showing (A) axial contrast section with the yellow arrow suggesting transverse colon involvement with adjacent bowel wall thickening, and (B) coronal section with the yellow arrow showing transverse colon involvement.

A physician referred the patient to the oncology department for further management. The treating surgeon discussed the case in the tumor board meeting, where it was decided that surgery would be followed by chemotherapy. Hence, the surgeon obtained consent from the patient and relatives after consultation. The patient underwent distal gastrectomy with gastrojejunostomy along with resection and anastomosis of a part of the transverse colon as the transverse colon was infiltrated by a pyloric mass (Figures 3, 4). Postoperatively, pharmacological treatment was started, including antibiotics and daily dressing. Alternate stapler removal was done, and on the 14th postoperative day, complete removal of the stapler was done. The histopathology report revealed well-differentiated adenocarcinoma of the stomach (mucin secreting) of diffuse type. Chemotherapy was given to the patient. Capecitabin-oxaliplatin chemotherapy (six cycles) was advised. The patient underwent the first cycle of chemotherapy with capecitabin-oxaliplatin. The physician recommended a post-chemotherapy complete blood count (CBC), and the reports were regular. The patient was stable and was discharged, with an advise to follow-up after three weeks with a repeat CBC and for the second cycle of chemotherapy.

## Discussion

Although SIT can be a hereditary disorder, the reasons behind it are not known. The present case of stomach carcinoma with SIT was operated by the open approach posing anatomical difficulties as the primary organs were a mirror image of their standard placement. Extensive lymph node dissection was conducted in the opposite way. A similar anatomical challenge was encountered by Yoshimoto et al., though they operated on the patient laparoscopically [5].

Cao et al. documented a patient with poorly differentiated adenocarcinoma with SIT at the gastroesophageal junction [2]. The present case had adenocarcinoma of the pylorus. Namikawa et al. in 2018 reported another patient with a spreading-type superficial stomach malignancy in a case with SIT. The present case had histopathology suggestive of a diffuse type of adenocarcinoma [7]. Gundeş et al. presented a case of well-differentiated adenocarcinoma with an early lesion, not similar to the present advanced and diffuse carcinoma [8]. Abbey et al. published a case of an elderly male with stomach malignancy with synchronous SIT who was operated on using Roux-en-Y reconstruction and distal gastrectomy with DaVinci robot assistance [9].

Considering difficulties in arriving at a diagnosis by histopathology, it is not always a gold standard investigation in a case of stomach carcinoma. Immunohistochemistry is needed as gastric leiomyoma and gastric schwannomas are important differential diagnoses, as observed by Mulita et al. in their study [10].

## Conclusions

The presence of primary gastric cancer in conjunction with situs inversus is a unique clinical scenario. Even though the simultaneous appearance of gastric mucinous adenocarcinoma and SIT is a rare anatomopathological entity, all clinicians should be familiar with making an appropriate and precise diagnosis that will lead to quick and right therapy. It has been frequently documented that operating in such cases can prove very challenging even for experienced physicians due to extensive anatomical variations. There is also extensive proof of treating such cases with surprisingly positive outcomes. A thorough preoperative screening with various imaging modalities should be included in the prerequisites for this condition to minimize morbidity and mortality and decrease the pathological burden on society, especially in developing countries. Numerous approaches can be successfully used to treat such patients, such as robotic surgeries, open surgeries, and laparoscopic surgeries.
